# Multifaceted and educational interventions to improve prescribing indicators in the Middle East and North Africa Region: a systematic review and meta-analysis

**DOI:** 10.1007/s11096-026-02107-1

**Published:** 2026-03-17

**Authors:** Muhammad Ilyas, Tawanda Chivese, Muhammad Abdul Hadi, Nondumiso Beauty Queeneth Ncube, Muhammad Naseem Khan

**Affiliations:** 1 Department of Population Medicine, College of Medicine, QU Health, Qatar University, PO Box 2713, Doha, Qatar; 2 Department of Science and Mathematics, School of Interdisciplinary Arts and Sciences, University of Washington Tacoma, Tacoma, WA 98402 USA; 3 Department of Clinical Pharmacy and Practice, College of Pharmacy, QU Health, Qatar University, Doha, Qatar; 4 Department of Community and Health Sciences, School of Public Health, University of the Western Cape, Cape Town, South Africa

**Keywords:** Antibiotic prescribing, Antimicrobial resistance, Educational intervention, Meta-analysis, Middle East, North Africa, Polypharmacy, Prescribing behavior, Rational prescribing, Systematic review, WHO/INRUD indicators

## Abstract

**Introduction:**

Rational prescribing is challenging due to global antibiotic resistance and widespread polypharmacy. Evidence on effective interventions to improve prescribing practices in MENA is limited.

**Aim:**

This systematic review and meta-analysis evaluated the effectiveness of multifaceted and educational interventions in improving WHO/INRUD prescribing indicators in the Middle East and North Africa (MENA).

**Method:**

We searched PubMed, Scopus, and CINHAL up to June 10, 2025, for experimental studies evaluating the effectiveness of multifaceted interventions on WHO/INRUD prescribing indicators. Searches were supplemented by screening reference lists of relevant reviews, first 20 pages of Google Scholar results, and ResearchRabbit.ai for citation-chaining to identify additional records. Primary outcomes included WHO/INRUD prescribing indicators: average number of drugs per prescription, percentage of encounters with an antibiotic prescribed, percentage of encounters with an injection prescribed, percentage of drugs prescribed by generics, and percentage of drugs prescribed from the essential medicines list. Methodological quality was assessed with the MASTER scale. Meta-analysis used a bias-adjusted inverse-variance heterogeneity model adjusted with quality score. Heterogeneity and publication bias were assessed using *I*^2^, the Doi plot, and LFK index.

**Results:**

Sixteen studies (seven RCTs, six pre-post, three quasi-experimental) from Iran, UAE, Egypt, Sudan, Lebanon, Saudi Arabia, and Palestine were included. Multifaceted interventions modestly reduced the average number of drugs per prescription (weighted mean difference WMD − 0.10, (95% CI − 0.18 to -0.02; *I*^2^ = 99.8%). There was a downward trend in the odds of prescriptions with antibiotics (OR 0.65; 95% CI 0.41 to 1.03, *I*^2^ = 93.5%) and injections (OR 0.93, 95% CI 0.82 to 1.04, *I*^2^ = 25.3%), though these did not reach statistical significance and confidence intervals included the possibility of no effect. Meta-analysis revealed extreme statistical heterogeneity (*I*^2^ up to 99.8%), and the GRADE certainty of evidence was low to very low for all outcomes.

**Conclusion:**

Multifaceted interventions in the MENA region demonstrate potential for modest improvements in prescribing indicators, though evidence certainty remains low to very low. With non-significant pooled effects for antibiotics and injections, these exploratory findings suggest that context-specific stewardship and prescribing quality programs can achieve targeted improvements, but also highlight the need for more locally led, rigorous research with longer follow-up to inform policy decisions.

**Supplementary Information:**

The online version contains supplementary material available at 10.1007/s11096-026-02107-1.

## Impact statements

- Multifaceted interventions that combine audit and feedback with education may reduce inappropriate antibiotic use, but their impact on polypharmacy, injections, and effectiveness across the Middle East and North Africa remains limited and uncertain.
- In MENA, multifaceted prescribing interventions achieve only modest, highly variable effects on prescribing habits. Given the low-to-very-low certainty of the evidence, current data are insufficient to confirm their effectiveness in reducing polypharmacy, antibiotic use, or injection use.
- Education-only approaches appear insufficient, whereas programs that integrate audit and feedback, stewardship, and organizational support yield better results but must be tailored to local health system constraints.
- These findings highlight the need for more locally led, rigorous research in the MENA region, with longer follow-up. Policy decisions should focus on context-sensitive stewardship and prescribing quality programs that are adapted to the unique challenges of regional health systems.

## Introduction

Irrational prescribing is a major public health issue globally and in the Middle East and North Africa (MENA), leading to increased healthcare costs, antimicrobial resistance (AMR), and poor patient outcomes [1]. Up to 50% of medications prescribed in the region are unnecessary or inappropriate, characterized by antibiotic overuse, polypharmacy, and the use of ineffective drugs [2]. AMR is associated with an estimated 4.5 million deaths annually [3, 4] while polypharmacy increases morbidity, mortality, and drug interactions among older adults [5] and drives wasteful healthcare spending and longer hospital stays [6–8]. In the Gulf Cooperation Council (GCC) and MENA, inappropriate medication use ranges from 12 to 88%, alongside a high burden of non-communicable diseases, rising AMR [9, 10], rapid health-system transformation [11], and cultural norms of self-medication [12–14].

To address this challenge, the WHO and the International Network for Rational Use of Drugs (INRUD), developed core prescribing indicators to benchmark and monitor performance. These include, fewer than two drugs prescribed per prescription, antibiotics in no more than 30% of encounters, injectables in no more than 20% of encounters, all drugs prescribed by generic name, and from an essential medicines list [15, 16]. These WHO/INRUD indicators provide a standardized framework to assess and compare prescribing quality across settings [17].

Multiple interventions can modify prescribing behavior, including educational materials, workshops, audit and feedback, electronic reminders, financial incentives, point-of-care testing, communication training, and mass media campaigns [18]. Single-component interventions are rarely sufficient. Multifaceted approaches, two or more coordinated strategies [19] like academic detailing with feedback or electronic tools combined with guidelines, education, and formulary restrictions [20], produce more sustainable behavioral change [21]. For example, combining clinician education with computer support can reduce inappropriate antibiotic prescribing by 4% to 34% [22], while adding patient-directed interventions can reduce it by 6 to 21% [23]. Multifaceted interventions have produced the largest reductions in inappropriate prescribing (73.2%) and diagnostic testing (70.4%), whereas education alone was the least effective [24]. Recent antimicrobial stewardship (ASP) intervention efforts in the region, including a Lean Six Sigma based program in the UAE, have reduced parenteral antimicrobial expenditure by 81.7% and overall antibiotic use by 54.2% [25]. Such initiatives, supported by strong leadership, show the region’s growing commitment to stewardship [26].

Despite international evidence, there is no comprehensive synthesis of how multifaceted and educational interventions affect prescribing indicators in the MENA context. Regional prescribing remains heavily influenced by a "policy–behavior gap," in which healthcare modernization efforts often clash with fragmented regulations and entrenched traditional norms [11]. Multifaceted programs are essential in MENA because they target prescriber behavior, organizational processes, and patient expectations. By mapping these objectives to WHO/INRUD indicators, this review provides a context-specific framework to improve prescribing quality and patient safety across the region [10, 14].

## Aim

This systematic review and meta-analysis aimed to assess the effectiveness of multifaceted and educational interventions in improving prescribing indicators in the Middle East and North Africa (MENA) region.

## Method

### Research framework

This review used the PICO framework: Population (P) included prescribers and patients in healthcare settings; Intervention (I) was multifaceted interventions to improve prescribing; Comparator (C) was usual care; Outcome (O) was prescribing indicators measured by WHO/INRUD.

### Study design

This systematic review and meta-analysis was prospectively registered in PROSPERO (CRD42024533958) and conducted in accordance with PRISMA guidelines [27].

### Data sources

We searched PubMed, Scopus, and CINAHL from inception to 10 June 2025, with no language restrictions. Supplementary sources included the first 20 pages of Google Scholar results, the reference lists of relevant reviews, and WHO/INRUD documents as additional gray literature. We also used ResearchRabbit.ai, a citation-based literature mapping tool, to identify articles from core studies and screened them using the same criteria.

### Search strategy

PICO elements (Population, Intervention, Comparator, Outcomes, and Study design) informed eligibility criteria, but population and comparison terms were deliberately omitted from the search to maximize sensitivity and avoid missing relevant MENA studies [28]. The strategy combined intervention and prescribing terms with a comprehensive list of MENA countries and regional terms. Full database search strings are provided in Supplementary Document 1.

### Procedure for study selection

All search results were imported into the Rayyan software to organize records, detect duplicates, and manage screening. Two reviewers (MI & TC) independently screened titles and abstracts, reviewed full texts, and assessed eligibility. Disagreements were resolved by a third reviewer (MNK).

### Inclusion criteria

Studies were eligible if they used any combination of interventions defined by the Effective Practice and Organization of Care (EPOC) taxonomy [18], including educational, informational, audit and feedback, decision support, system-oriented, multimodal, or communication strategies. Consistent with EPOC guidance, multifaceted interventions were defined as those combining two or more distinct components. These were the primary focus of this review, whereas single-component educational interventions were included as contextual comparators. Eligible studies were required to report at least one WHO/INRUD prescribing indicator (or provide sufficient data to derive one), with no restrictions on language. Conversely, observational studies, animal models, and research lacking specific prescribing indicators were excluded from the analysis.

### Outcomes

Primary outcomes were WHO/INRUD indicators: average number of drugs per encounter, percentage of encounters with an antibiotic, percentage of encounters with an injection, percentage of drugs prescribed generically, and percentage from the essential medicines list. The secondary outcome was the appropriateness of treatment, particularly antibiotics, defined as the percentage of prescriptions compliant with guideline-recommended choices, doses, and durations.

### Data extraction

Two reviewers (MI & TC) independently extracted data using a structured Excel form, including study design, sample size, population, country, setting, intervention, comparison, indicators, outcomes, duration, and results. Disagreements were resolved through discussion, with arbitration by a third reviewer (MNK) as needed. To ensure a uniform comparator and account for inconsistent baseline reporting, only post-intervention data were extracted for quantitative synthesis. Follow-up durations were also documented for each study to assess the time horizon of reported effects.

### Quality assessment

Study quality was assessed using the Methodological Standard for Epidemiological Research (MASTER) scale, which provides a unified bias assessment across randomized and quasi-experimental designs and enables direct comparisons across studies. MASTER scale includes 36 safeguards in seven domains (equal recruitment, retention, ascertainment, implementation, prognosis, analysis, and temporal precedence) [29]. Safeguards were scored as 1 (present) or 0 (absent), summed for an overall score to rank studies and inform bias-adjusted meta-analyses. Two reviewers (MI & TC) independently applied MASTER, and disagreements were resolved by a third reviewer (MNK).

### Data synthesis and analysis

The meta-analysis used the inverse-variance heterogeneity model, incorporating MASTER quality scores [30], which maintain valid coverage and a lower false-positive rate than random-effects models in the presence of extreme heterogeneity and bias. Pooled effects were calculated as Odds Ratios (ORs) for antibiotic and injection proportions (dichotomous) and Mean Differences (MD) for drugs per encounter (continuous). Pooling was done to estimate the regional direction and distribution of effects rather than a single common effect. To maintain integrity, pooling was restricted to study means or proportions; outcomes expressed as Defined Daily Doses (DDD) were synthesized narratively. Heterogeneity was assessed using Cochran’s Q statistic and the *I*^2^ statistic (25%, 50%, 75% for low, moderate, and high) [31]. Robustness was tested through leave-one-out and quality-based sensitivity analyses, the latter excluding studies with the lowest MASTER scores. To address the conceptual diversity of the included studies, we performed exploratory subgroup analyses comparing intervention types (educational vs. multifaceted/structural), study design (RCTs vs. quasi-experimental), healthcare setting (primary care vs. hospitals), and region (GCC vs. other MENA countries). While our primary focus was on multifaceted interventions, single-component studies were included to provide a comparative baseline for multifaceted approaches. Publication bias was evaluated with the Doi plot and LFK index [32], which are more reliable than funnel plots or Egger regression [33]. LFK values between ± 1 indicate minor asymmetry, whereas ± 2 indicates significant asymmetry [34].

### Certainty of evidence assessment

Certainty of evidence for outcomes in the meta-analyses was assessed using the Grading of Recommendations, Assessment, Development, and Evaluation (GRADE) framework, resulting in classifications of high, moderate, low, or very low certainty. This approach is widely accepted and used to assess the evidence level based on five domains: risk of bias, inconsistency, indirectness, imprecision, and publication bias.

## Results

### Search results

**Fig. 1:**
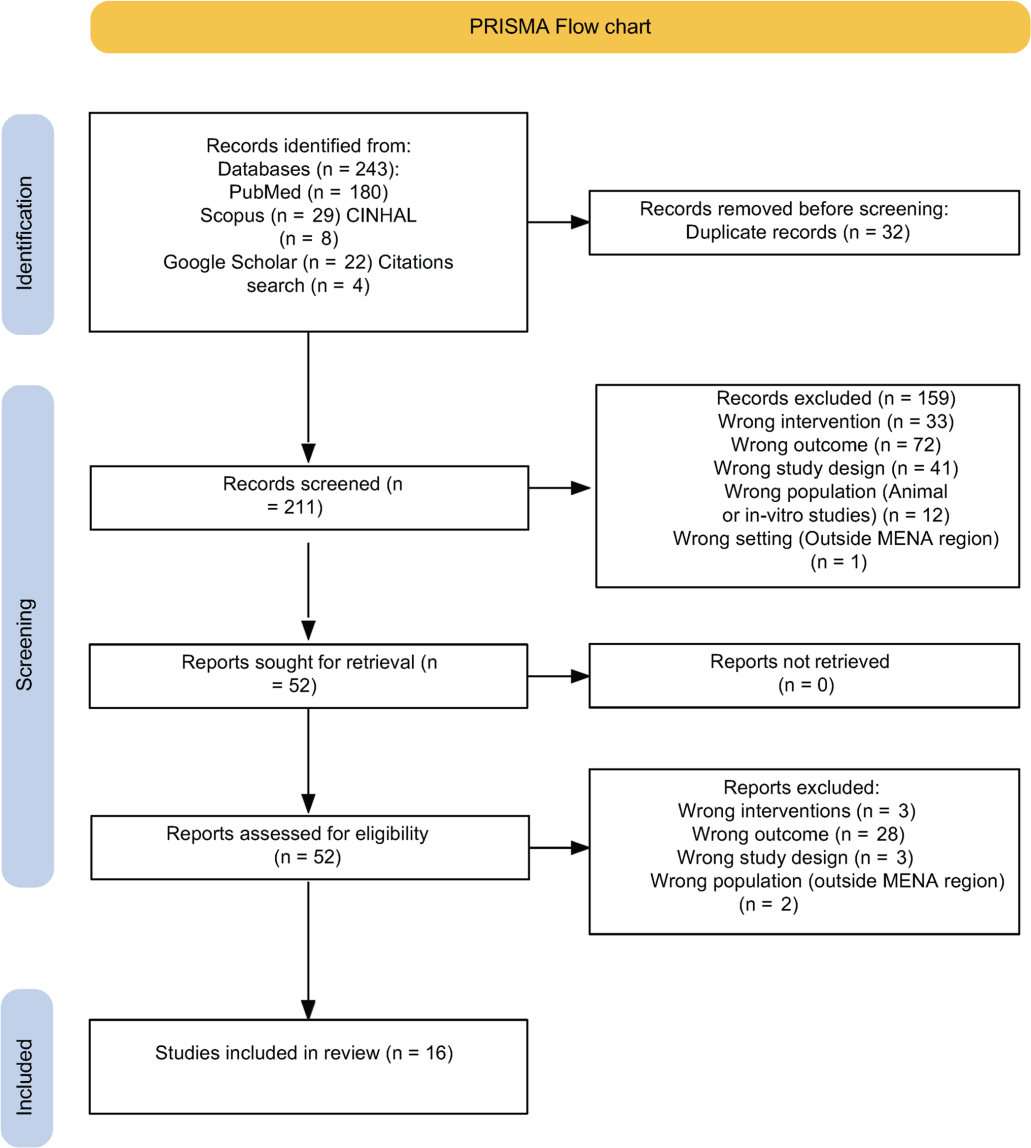
PRISMA Flow chart

The search identified 243 records; after removing 32 duplicates, 211 titles and abstracts were screened, with 159 excluded. Of 52 full texts assessed, 36 were excluded (wrong intervention *n* = 3, wrong design *n* = 3, wrong outcome *n* = 28, wrong setting *n* = 2). Sixteen studies met the inclusion criteria (see Fig. 1).

### Characteristics of included studies

Of the 16 included studies, seven were randomized controlled trials, six were pre-post, and three were quasi-experimental. Settings included primary care centers [35–41], hospitals [40, 42–46], private clinics [47, 48], and specialized institutions [49, 50]. Five studies were from Iran [35, 37, 38, 46, 48], five from GCC countries (UAE and Saudi Arabia) [41–43, 45, 50], two each from Egypt [40, 44] and Sudan [36, 39], and one each from Lebanon [47] and Palestine [49]. Full characteristics are summarized in Table 1 and Supplementary Table 1.

**Table 1:** Characteristics of the included studies

Author & Country	Study Design	Study Setting	Intervention Details	Outcome indicators
Awad [36], Sudan	cRCT	Health centers	A & F, A & F + seminar, A & F + AD	% of encounters with an antibiotic prescribed
Chehabeddine [47], Lebanon	RCT	Private dental clinics	Educational Intervention (antibiotic overuse, AMR, guidelines for appropriate treatment)	% of encounters with an antibiotic prescribed
Elhabil [49], Palestine	Pre and Post	Specialized Pediatric Hospital	Performing medication use assessments, and consulting with cardiologists to resolve inappropriate prescribing	Mean number of drugs prescribed per encounter % encounters with antibiotics prescribed % encounters with injection prescribed % of drugs prescribed by generic % of drugs prescribed from the essential drug list
Esmaily [37], Iran	cRCT	GPs clinics	Educational training Self-learning educational materials	Mean number of drugs prescribed per encounter % encounters with antibiotics prescribed % encounters with injection prescribed
Garjani [48] Iran	RCT	Public and private physicians’ clinic	Interactive group discussion on rational prescribing & Routine practices	Mean number of drugs prescribed per encounter % encounters with antibiotics prescribed % encounters with injection prescribed % of drugs prescribed by generic % of drugs prescribed from the essential drug list
Hasan [41], Sharjah, UAE	Pre and Post	Primary Health Care Centers	ACC guidelines	Mean number of drugs prescribed per encounter % encounters with antibiotics prescribed % encounters with injection prescribed % of drugs prescribed by generic % of drugs prescribed from the essential drug list
Kandeel [40], Egypt	Pre & Post	Primary healthcare centers, hospitals, private pharmacies	Educational material, 5-days Training course, Communication and Social media campaign using Facebook and YouTube channel	% of encounters with an antibiotic prescribed
Ibrahim [44], Egypt	Pre and Post	Physicians	Distributing antibiotic guidelines and holding workshops activities directed towards rational drug use	Mean number of drugs prescribed per encounter % encounters with an antibiotics % encounters with an injection % of drugs prescribed by generic % of drugs prescribed from the essential drug list
Soleymani [35], Iran	RCT	Outpatients' clinic	RA&F NA&F PEM	Mean number of drugs prescribed per encounter % encounters with an antibiotic prescribed % encounters with an injection prescribed
Salehi [46] Iran	Pre and Post	Hospital outpatients Emergency department	20-min educational video regarding appropriate indication, dose, and duration of antibiotics	% encounters with an antibiotic prescribed
Eltayeb [39], Sudan	cRCT	Government health centers	A&F, A&F + seminars + Prescribing guidelines, A&F + AD + prescribing guideline No Intervention (Control)	% of encounters with appropriate antibiotics
Nejad [38], Iran	RCT	Physicians	Feedback through TPLs Feedback through STM	Define daily dose
Alnajjar [50], KSA	Quasi-experimental	Rehabilitation specialist hospital	ASP, Audits, & feedback, Restrictive interventions, pre-authorization of restricted antibiotics	Preprocedural prophylaxis post-procedure prophylaxis
Alshehhi [45], UAE	Pre and Post	Secondary care government hospital	Implementation of ASP	% of encounters with appropriate antibiotics
Elnajjar UAE [43]	Quasi-experimental	Tertiary care hospital	ASP	Define daily dose
Gulam UAE [42]	Quasi-experimental	Hospital	Pharmacist-led prospective audit and feedback	Length of therapy

AB—Antibiotic; ACC—Antibiotic Control Committee; A&F—Audit and Feedback; AMR—Antimicrobial Resistance; ASP—Antimicrobial Stewardship Program; DDD—Defined Daily Dose; Edu—Educational; GL—Guideline; Med—Medication; NA&F—New-design Audit and Feedback; PEM—Printed Educational Materials; RA&F—Routine Audit and Feedback; STM—Short Text Messages; TPLs—Traditional Postal Letters; PHC—Primary Health Care

MD—Mean Difference; SD—Standard Deviation; CI—Confidence Interval; cRCT—Cluster Randomized Controlled Trial; RCT—Randomized Controlled Trial

Outcomes used in meta-analysis: Mean number of drugs prescribed per encounter; % encounters with an antibiotic; % encounters with an injection

Outcomes used in narrative analysis: % of drugs prescribed by generic; % of drugs prescribed from the essential drug list; percentage of encounters with appropriate antibiotics

### Description of the quality of included studies

**Fig. 2:**
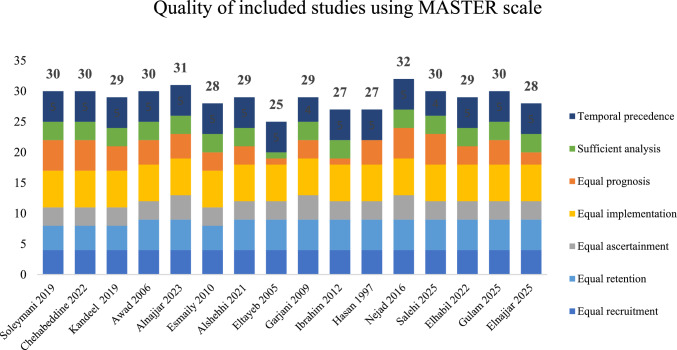
Quality assessment of included studies using the MASTER scale

On average, studies met 29 of 36 MASTER safeguards (range 25–32), indicating generally good quality. However, none reported methods for managing missing data, blinding, or allocation concealment. Five studies described randomization. Most showed adequate recruitment, retention, and analysis (see Fig. 2).

### Effectiveness of multifaceted and educational interventions on the average number of drugs per encounter

**Fig. 3:**
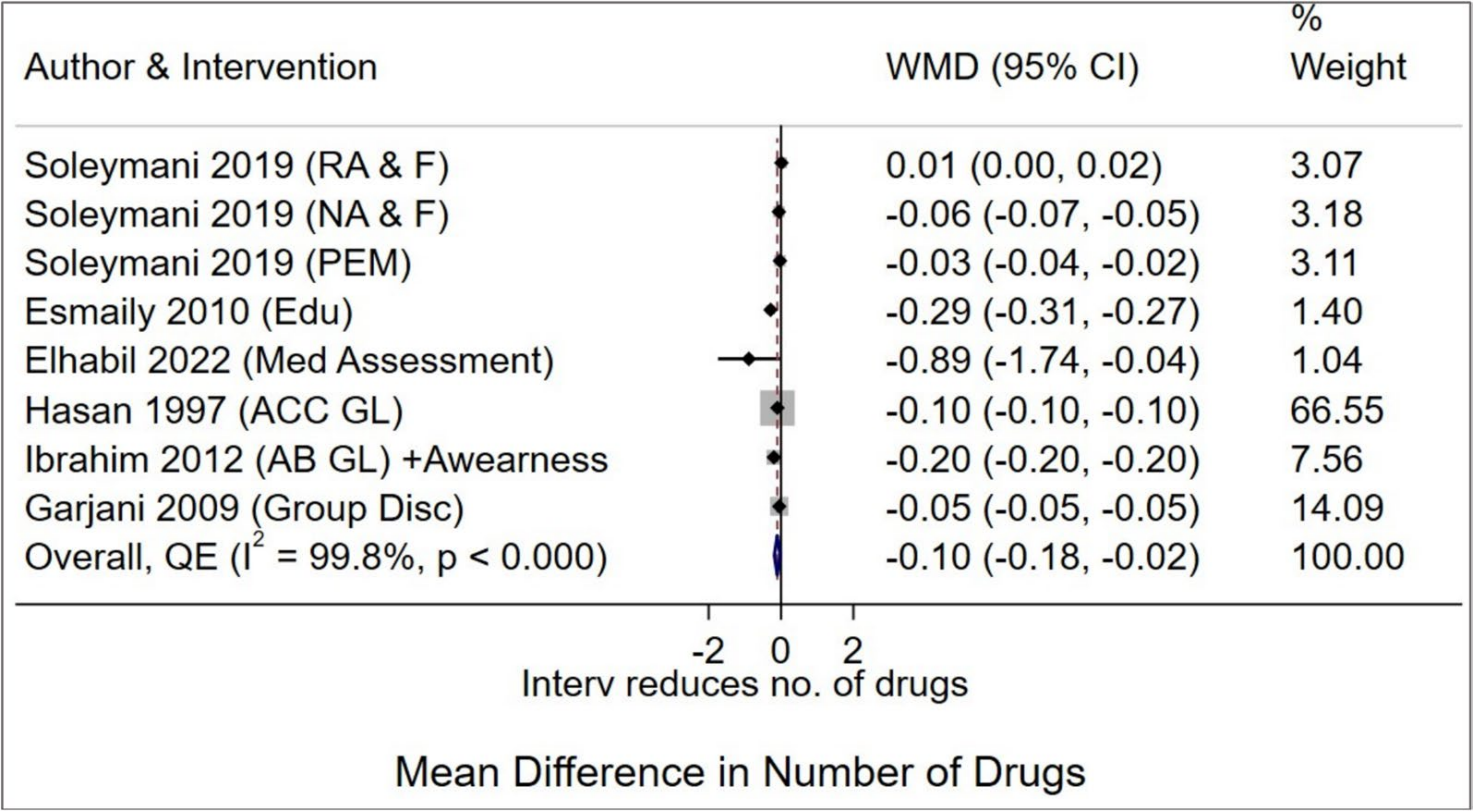
Forest plot for the effectiveness of multifaceted and educational interventions on the average number of drugs per encounter

Six studies [35, 37, 41, 44, 48, 49] assessed the effectiveness of multifaceted interventions on the mean number of drugs per encounter. Pooled analysis showed a modest reduction in the average number of medications per encounter (WMD -0.10 (95% CI -0.18 to -0.02), with very high heterogeneity (*I*^2^ = 99.8%, *p* < 0.001) (Fig. 3). Neither leave-one-out analysis (WMD -0.09 to –0.11; Supplementary Fig. 1) nor excluding studies with the lowest MASTER scores significantly altered the effect size. Doi plot and LFK index indicated substantial asymmetry, suggesting publication bias or small-study effects (Supplementary Fig. 2). Exploratory subgroup analyses by setting, design, intervention type, and region failed to reduce heterogeneity (*I*^2^ = 99.8%) or reveal significant differences between groups. (Supplementary Table 3).

### Effectiveness of multifaceted and educational interventions on the percentage of encounters with an antibiotic prescribed

**Fig. 4:**
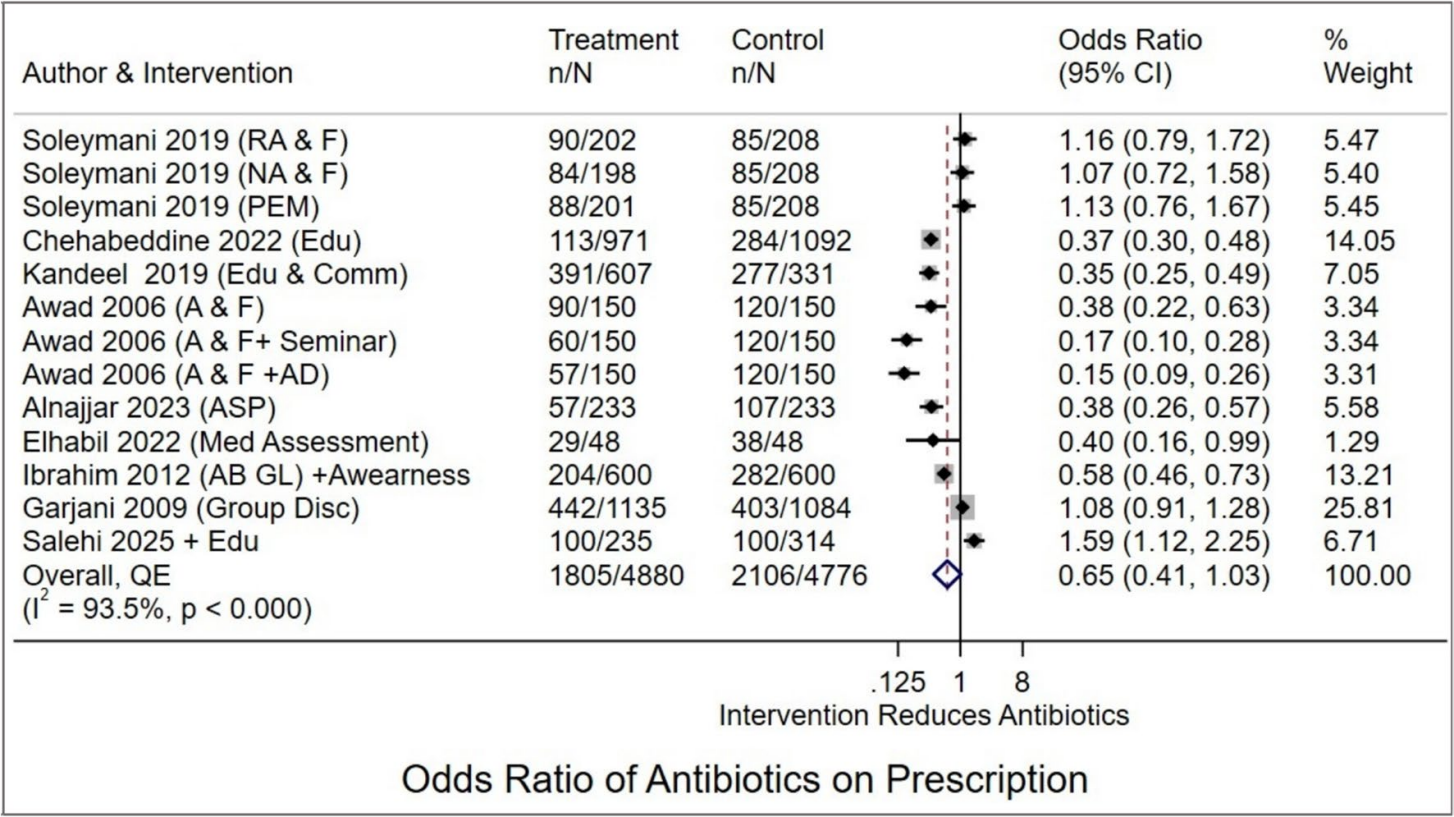
Forest plot showing the effectiveness of multifaceted and educational interventions on the percentage of encounters with an antibiotic prescribed

Eleven studies [35–37, 40, 44, 46–50] assessed the effectiveness of multifaceted interventions on the percentage of encounters with an antibiotic prescribed. Pooled odds ratio (OR) showed a nonsignificant downward trend (OR 0.79, 95% CI 0.50 to 1.24, *I*^2^ = 95.7%, *p* =  < 0.001) (Supplementary Fig. 3). Sensitivity analysis identified two influential studies [37, 41] (Supplementary Fig. 4); excluding them produced a similar downward trend that did not reach statistical significance (OR 0.65, 95% CI 0.41 to 1.03;  *I*^2^= 93.5%, *p* < 0.001) (Fig. 4). Excluding studies with the lowest MASTER scores also produced no meaningful results. Doi plot and LFK index indicated minor asymmetry, suggesting slight publication bias (Supplementary Fig. 5). Subgroup analysis showed larger effects (53%) for multifaceted strategies (OR 0.48, 95% CI 0.24 to 0.93) compared to education-only (OR 0.73, 95% CI 0.43 to1.25), but this difference was not statistically significant (*p* = 0.332). Because heterogeneity remained high (*I*^2^ = 93.5%), this trend should be viewed as hypothesis-generating rather than a definitive comparison of intervention types. No other significant differences were observed by design, setting, or region (Supplementary Table 4).

### Effectiveness of multifaceted and educational interventions on the percentage of encounters with an injection prescribed

**Fig. 5:**
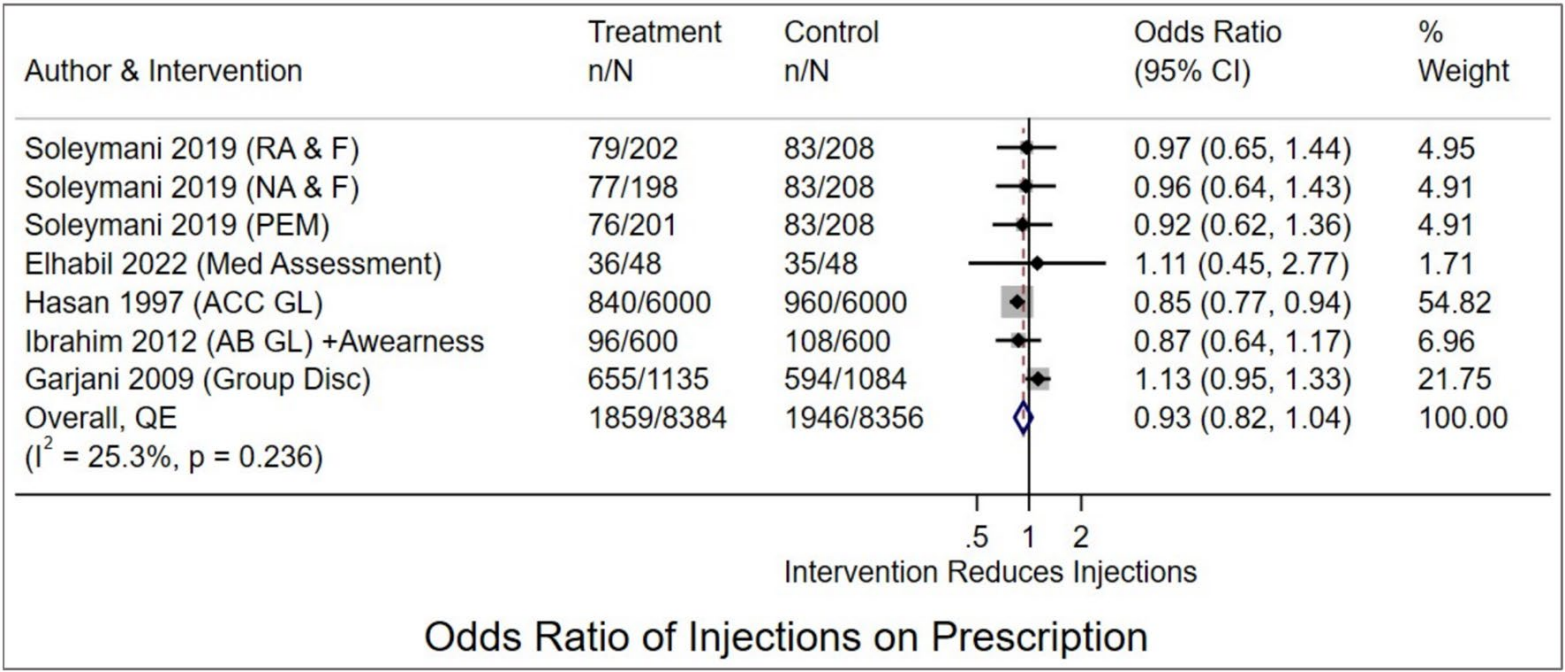
Forest plot showing the effectiveness of multifaceted and educational interventions on the percentage of encounters with an injection prescribed

Six studies [35, 37, 41, 44, 48, 49] evaluated the effectiveness of multifaceted interventions on the percentage of encounters with an injection prescribed. Pooled analysis showed a potential 15% reduction (OR 0.85, 95% CI 0.74 to 0.97; *I*^2^ = 58.8%, *p* = 0.017), with moderate heterogeneity (Supplementary Fig. 6). However, excluding one influential study [37] (Supplementary Fig. 7) rendered the effect non-significant and reduced heterogeneity (OR 0.93, 95% CI 0.82 to 1.04, *I*^2^ = 25.5%, *p* = 0.236) (Fig. 5). Sensitivity analyses excluding studies with the lowest MASTER scores showed no meaningful changes in estimates. Doi plot and LFK index showed major asymmetry, suggesting potential publication bias or small-study effects (Supplementary Fig. 8). Subgroup analysis by study design showed greater reductions in quasi-experimental studies (OR 0.86, 95% CI 0.78 to 0.96) but not in RCTs (OR 1.06, 95% CI 0.92 to 1.21; *p* = 0.016). Effects were larger for multifaceted than education-only interventions (OR 0.88, 95% CI 0.80 to 0.93 vs OR 1.04, 95% CI 0.87 to1.24; *p* = 0.102), although this difference was not statistically significant. However, given the limited number of studies, this finding is prone to bias and should be interpreted cautiously. No significant differences were found by setting or region (Supplementary Table 5).

### Prescriptions of generics and drugs from the essential medicines list (EML)

Three studies reported on generic prescribing and EML use [37, 44, 49]. In two studies, generic prescribing remained at 0% and EML use at 100% before and after the intervention. Only one study found a significant increase in generic prescribing (97.2% vs 72%, *p* = 0.002) and a non-significant rise in EML use (99.3% vs 95.2%, *p* = 0.152) [49].

### Effectiveness of multifaceted and educational interventions on the percentage of encounters with appropriate antibiotics

Four studies assessed the effectiveness of multifaceted and educational interventions on appropriate antibiotic use, with mixed results. One Lebanese study reported a significant difference for invasive dental procedures (55.8% vs 13.6%, *p* = 0.001), but no difference for infectious disease [47].

Another showed incremental gains: audit and feedback alone (mean difference 3.2, 95% CI 0.3 to 6.1, *p* = 0.034), increasing with seminars/guidelines (mean difference 9.6, 95% CI 6.7 to 12.5, *p* < 0.001), and peaking with academic detailing (mean difference 11.2, 95% CI 8.3 to14.1, *p* < 0.001) [39]. An ASP paired with education, and audits and feedback reduced pre- and post-procedure prophylaxis (45.9% to 24.46%; *p* < 0.001) and (16.7% to 1.2%; *p* < 0.001) [50], whereas another ASP study showed a slight decline in appropriate antibiotic selection (55.3% to 52.1%) [45] (Supplementary Table 1).

### Evidence grading

Certainty of evidence was downgraded for all outcomes because most studies were non-randomized and prone to bias and confounding. As a result, certainty was rated low for the average number of drugs and very low for antibiotic and injection outcomes, indicating that these effect estimates are highly uncertain (Supplementary Table 2).

## Discussion

This systematic review, the first to synthesize multifaceted and educational interventions in the MENA region, found modest reductions in the average number of drugs, antibiotic use, and injections. Given the extreme heterogeneity and low-to-very-low GRADE certainty, these findings represent exploratory regional trends rather than definitive effectiveness.

Reductions in drugs per encounter were smaller than those reported in international meta-analyses, which have reported larger reductions in polypharmacy (MD − 0.57; 95% CI − 0.80 to − 0.34) [51] and prescribed medications (SMD − 0.25; 95% CI − 0.38 to − 0.13) [52]. This likely reflects higher baseline prescribing, implementation challenges and weaker behavior-change in MENA. While high-income countries integrate interventions into digital and regulatory frameworks, MENA and other LMICs face workforce shortages, fragmented care, and limited data systems. Substantial heterogeneity, seen globally (*I*^2^ = 79% to 90%, vs 99.7% here), is intrinsic to complex interventions where system support for behavior change varies widely [53].

Our findings on antibiotic prescribing align with global antimicrobial stewardship programs (ASPs) evidence, where audit and feedback typically reduce antibiotic use by 15 to 30% [54, 55]. Pharmacist-led strategies also show significant promise in improving selection and reducing volume [56, 57]. In this review, multifaceted strategies generally outperformed education-only interventions, supporting the need to address multiple determinants of prescribing simultaneously, including knowledge, habits, norms, patient expectations, and organizational constraints [58]. While education alone often fails due to a lack of prescriber autonomy or system support, multifaceted strategies address diverse barriers through synergistic mechanisms. Audit and feedback promote accountability, while structural changes, such as stewardship committees, create the environmental conditions necessary to sustain new habits [59].

The modest reduction in injection prescribing is a novel finding, as no previous meta-analyses have examined this outcome, but sensitivity analysis indicated that it was largely driven by one influential study and may be biased. Notably, no improvement was seen in generic prescribing or EML adherence. These structural indicators exhibited "all-or-nothing" patterns, suggesting that they are determined by procurement policies and national formularies rather than individual prescribers’ choices. While multifaceted interventions may improve clinicians' capabilities, they are unlikely to close this “policy–behavior gap” without supply-chain reforms and regulatory support for generic substitution.

This regional evidence is limited by pre-post and quasi-experimental designs with limited reporting of randomization, allocation concealment, and blinding, and is vulnerable to secular trends and increased risk of bias. To mitigate these risks, we applied the IVhet model using MASTER quality scores. Since excluding the weakest designs did not alter outcomes, low certainty appears to be a systemic feature of the regional literature.

Exploratory subgroup and sensitivity analyses did not explain the extreme heterogeneity in polypharmacy and antibiotics (*I*^2^ > 93%, *p* > 0.05), indicating that most variability reflects genuine differences in populations, settings, and implementation rather than chance. Consistent with the nature of complex interventions, this heterogeneity likely arises from clinical diversity, methodological differences, and variation in implementation fidelity across MENA health-care systems [59, 60]. Despite this, we retained the meta-analysis for three reasons. First, most studies had consistent. direction of effect towards reduction in polypharmacy, antibiotic use, and injection use, even with wide confidence intervals crossing the null. Therefore, pooling summarizes the regional trends and distribution of intervention effects, rather than estimating a precise single effect size. Second, outcomes were WHO/INRUD core indicators with standardized definitions, which reduce conceptual heterogeneity; and third, methodological guidance indicates that IVhet models can provide useful average effects for complex service-delivery interventions when interpreted cautiously and accompanied by sensitivity analyses [61]. Accordingly, pooled estimates are treated as exploratory summaries of overall direction with GRADE evidence as low or very low. These trends identify priority areas for future research but are insufficient to prove that one intervention is more effective than another.

### Strengths and limitations

This is the first meta-analysis to synthesize multifaceted and educational interventions for all WHO/INRUD prescribing indicators in the MENA region. We used a highly sensitive search strategy, supplemented with citation chaining, and utilized the MASTER quality tool and bias-adjusted models for robust analysis. By focusing on the "patient-provider dyad," provides a culturally relevant evidence base to improve prescribing practices in the region.

However, most studies were from Iran and the UAE, limiting generalizability to lower-income or conflict-affected settings. The scarcity of high-quality RCTs, very high residual heterogeneity (*I*^2^ > 93.5%), short follow-up in some studies, and “Very Low” GRADE ratings, particularly for structural indicators shaped by rigid national policies, limit confidence in applying pooled effects across diverse financing models and cultural norms.

### Significance of the findings

This meta-analysis shows that multifaceted and educational interventions can reduce polypharmacy, promote appropriate antibiotic use, and reduce unnecessary injections, directly benefiting patient safety, AMR control, and healthcare costs. However, substantial heterogeneity indicates that no single multifaceted intervention is sufficient; effects depend on how components are tailored to local workflows, regulations, and prescribing norms, and on the quality of implementation and policy support for generic and essential list use.

## Conclusion

Multifaceted interventions that combine educational interventions, audit and feedback, academic detailing, and organizational restructuring (e.g., ASP) are potentially more effective than education alone in improving prescribing indicators in MENA, but the certainty of evidence is low to very low, so these findings require cautious interpretation. These strategies may enhance clinicians’ capabilities, motivation, and work environments, helping to overcome cultural and structural barriers, yet their impact and sustainability remain uncertain without stronger trials. Future research should prioritize cluster-randomized trials with longer follow-up (≥ 12 months) and process evaluations to assess implementation fidelity. Ultimately, closing the "policy–behavior gap" requires integrating stewardship with structural and regulatory reforms to ensure sustainable impact across the MENA region.

## Supplementary Information

Below is the link to the electronic supplementary material.

Supplementary file1 (DOCX 1100 KB)

## Data Availability

Data are available upon request from the corresponding author.
