# Supplementary material for: Multifaceted and educational interventions to improve prescribing indicators in the Middle East and North Africa Region: a systematic review and meta-analysis
Source: Int J Clin Pharm. 2026 Mar 17;48(3):710–22. doi: 10.1007/s11096-026-02107-1 (PMC13176026; doi:10.1007/s11096-026-02107-1)
Supplement: Supplementary file 1 — Supplementary file1 (DOCX 1100 KB) [file 11096_2026_2107_MOESM1_ESM.docx]

**Supplementary File:**

Title: Multifaceted and educational interventions to improve prescribing indicators in the Middle East and North Africa Region: a systematic review and meta-analysis

Authors:

Muhammad Ilyas

Department of Population Medicine, College of Medicine, QU Health, Qatar University, Doha, Qatar

Email: [mi2211197@qu.edu.qa](mailto:mi2211197@qu.edu.qa)

ORCID: <https://orcid.org/0000-0001-9696-2084>

Tawanda Chivese

Department of Science and Mathematics, School of Interdisciplinary Arts and Sciences, University of Washington Tacoma, Washington, WA98402, U.S.A.

Email: [tchivese@uw.edu](mailto:tchivese@uw.edu)

ORCID: <https://orcid.org/0000-0001-6621-6144>

Muhammad Abdul Hadi

Department of Clinical Pharmacy and Practice, College of Pharmacy, QU Health, Qatar University, Doha, Qatar

Email: [mabdulhadi@qu.edu.qa](mailto:mabdulhadi@qu.edu.qa)

ORCID: <https://orcid.org/0000-0003-0108-7833>

Nondumiso Beauty Queeneth Ncube

Department of Community and Health Sciences, School of Public Health, University of the Western Cape, Cape Town, South Africa

Email: [nncube@uwc.ac.za](mailto:nncube@uwc.ac.za)

ORCID: <https://orcid.org/0000-0003-0478-9523>

**Corresponding author**

Muhammad Naseem Khan

Department of Population Medicine, College of Medicine, QU Health, Qatar University, Doha, Qatar, PO Box 2713

[naseem@qu.edu.qa](mailto:naseem@qu.edu.qa)

ORCID: <https://orcid.org/0000-0002-0484-0985>

**Search strategy for the different databases**

**PubMed:**

**#1 Interventions**

Education OR behavior* OR "audit and feedback" OR "point-of-care tests" OR "educational materials" OR "educational meetings" OR "educational outreach visits" OR reminder OR "financial interventions" OR "communication strategies" OR "patient education" OR "shared decision making" OR "mass media campaigns" OR "delayed prescription strategy" OR stewardship OR "multifaceted intervention" OR "complex intervention" OR “combined strategies” OR “Antibiotic stewardship”

**#2 Outcome**

"Prescribing indicators" OR prescribe* OR "prescription pattern" OR "prescribing pattern" OR "prescribing practice" OR "Drugs Prescribed" OR "Antibiotic prescribing indicators" OR "Injection prescription" OR "injectable prescription" OR "Essential Drug List" OR "Generic Drug" OR polypharmacy

**#3 Study design**

“Clinical trial” OR “Randomized controlled trial” OR “Randomized trial” OR “Quasi-Experimental” OR “Quasi Experimental” OR Experimental OR “non-randomized” OR “pre & post” OR “pre and Post” OR “Interrupted time series” OR “quasi-randomised trial”

**#4 Region**

“Middle east” OR MENA OR “North Africa” OR GCC OR “Gulf cooperation council” OR Bahrain OR Kuwait OR Oman OR Qatar OR “Saudi Arabia” OR “United Arab Emirates” OR UAE OR “Algeria” OR “Djibouti” OR “Egypt” OR “Iran” OR Morrocco OR Jordon OR Lebanon OR Syria OR Tunisia OR Yemen OR Libya OR Somalia OR Palestine OR Sudan

**#5 Exclusion**

“Case series” OR “Cross sectional” OR “case control” OR “Cohort study” OR retrospective OR “Systematic Review” OR “Chart Review” OR “scoping review” OR “literature review" OR “meta-analysis”

#1 AND #2 AND #3 AND #4 NOT #5

**SCOPUS**

**#1 Interventions**

#1 TITLE-ABS-KEY ( ( "educational intervention*" OR "behaviour change" OR "audit and feedback" OR "point-of-care test*" OR "educational material*" OR "educational meeting*" OR "educational outreach visit*" OR remind* OR "financial intervention*" OR "communication strateg*" OR "patient education" OR "improved communication" OR "shared decision making" OR "mass media campaign*" OR "delayed prescription strateg*" OR stewardship OR "multifaceted intervention*" OR "complex intervention*" OR “combined strategie*” OR “Antibiotic stewardship” ) )

**#2 Outcome**

#2 TITLE-ABS-KEY ((“Prescribing indicators" OR prescribe* OR "prescription pattern" OR "prescribing pattern" OR "prescribing practice" OR "Drugs Prescribed" OR "Antibiotic prescribing indicators" OR "Injection prescription" OR "injectable prescription" OR "Essential Drug List" OR "Generic Drug" OR polypharmacy)

**#3 Study design**

#3 TITLE-ABS-KEY ((“Clinical trial" OR "Randomized controlled trial" OR "Randomized trial" OR "Quasi-Experimental" OR "Quasi Experimental" OR experimental OR "Pre and Post" OR "non-randomized" OR "pre & post" OR "Interrupted time series" OR “quasi-randomised trial”))

**#4 Region**

TITLE-ABS-KEY ((“Middle east” OR MENA OR “North Africa” OR GCC OR “Gulf cooperation council” OR Bahrain OR Kuwait OR Oman OR Qatar OR “Saudi Arabia” OR “United Arab Emirates” OR UAE OR “Algeria” OR “Djibouti” OR Egypt OR Iran OR Morrocco OR Jordan OR Lebanon OR Syria OR Tunisia OR Yemen OR Libya OR Somalia OR Palestine OR Sudan)

**#5 Exclusion**

TITLE-ABS-KEY (“Case series" OR "Cross sectional" OR "case control" OR "Cohort study" OR "retrospective" OR "Systematic Review" OR " chart" OR " scoping" OR "meta-analysis”)

#1 AND #2 AND #3 AND #4 NOT #5

**CINAHL**

**#1 Interventions**

"( ( "educational intervention*" OR "behavior change" OR "audit and feedback" OR "point-of-care test*" OR "educational material*" OR "educational meeting*" OR "educational outreach visit*" OR remind* OR "financial intervention*" OR "communication strateg*" OR "patient education" OR "improved communication" OR "shared decision making" OR "mass media campaign*" OR "delayed prescription strateg*" OR stewardship OR "multifaceted intervention*" OR "complex intervention*" OR “combined strategie*” OR “Antibiotic stewardship” ) )

**#2 Outcome**

((“Prescribing indicators" OR prescribe* OR "prescription pattern" OR "prescribing pattern" OR "prescribing practice" OR "Drugs Prescribed" OR "Antibiotic prescribing indicators" OR "Injection prescription" OR "injectable prescription" OR "Essential Drug List" OR "Generic Drug" OR polypharmacy))

**#3 Study design**

((“Clinical trial" OR "Randomized controlled trial" OR "Randomized trial" OR "Quasi-Experimental" OR "Quasi Experimental" OR experimental OR "Pre and Post" OR "non-randomized" OR "pre & post" OR "Interrupted time series" OR “quasi-randomised trial”))

**#4 Region**

((“Middle east” OR MENA OR “North Africa” OR GCC OR “Gulf cooperation council” OR Bahrain OR Kuwait OR Oman OR Qatar OR “Saudi Arabia” OR “United Arab Emirates” OR UAE OR “Algeria” OR “Djibouti” OR Egypt OR Iran OR Morrocco OR Jordan OR Lebanon OR Syria OR Tunisia OR Yemen OR Libya OR Somalia OR Palestine OR Sudan))

#1 AND #2 AND #3 AND #4

**Supplementary Table 1: Characteristics and results of the included studies**

| **Author & Country** | **Study Design** | **Study Setting** | **Intervention Target** | **Duration (Months)** | **Intervention Sample** | **Control Sample** | **Intervention Details** | **Outcome indicators** | **Summary findings** |
| --- | --- | --- | --- | --- | --- | --- | --- | --- | --- |
| Alnajjar  2023 [50],  KSA | Quasi-experimental | Rehabilitation specialist hospital | Physicians | 12 | 233 | 233 | ASP,  Audits, & feedback, Restrictive interventions, pre-authorization of restricted antibiotics | None | Preprocedural prophylaxis:  Pre vs post:  45.9% vs 24.5%, (p<0.001, chi-square test) post-procedure prophylaxis:  16.7% vs 1.2%, (p<0.001, chi-square test) |
| Alshehhi  2021[45],  UAE | Pre and Post | Secondary care government hospital | Surgeons | 2 | 347 | 146 | Implementation of ASP | None | Selection of appropriate antibiotic:  Pre vs Post ASP implementation:  55.3% vs 52.1%  Selection of inappropriate antibiotic:  44.7% vs 47.9% |
| Awad  2006 [36],  Sudan | cRCT | Health centers | Physicians | 3 | 600 | 600 | A & F,  A & F + seminar,  A & F+ AD | % of encounters with an antibiotic prescribed | % of encounters with antibiotics prescribed: A&F vs control: -60% vs 80%  A&F + seminar vs control: 40% vs 80% A&F + AD vs control: 38% vs 80%  % of inappropriate antibiotics encounters  A&F. vs control: 38% vs 48%  A&F + seminar vs control: 21% vs 48% A&F + AD vs control: 15% vs 48% |
| Chehabeddine 2022 [47], Lebanon | RCT | Private dental clinics | Dentists | 2 | 971 | 1092 | Educational Intervention (antibiotic overuse, AMR, guidelines for appropriate treatment) | % of encounters with an antibiotic prescribed | Invasive oral and dental procedures  Mean % of antibiotics prescribed  Intervention vs control  9.0±14.3 vs 33.5±32.5, p=0.001  % of encounters with antibiotic, compliant with indication  55.8% vs 13.6%, p<0.001  Diseases of infectious origin  Mean % of antibiotics prescribed  74.7 ± 31.1 vs 79.0 ± 27.3  % of encounters with antibiotic compliant with indication  20.9% vs 22.3%, p=0.690 |
| Elhabil  2022 [49],  Palestine | Pre and Post | Specialized Pediatric Hospital | Cardiologists | 12 | 48 | 48 | Performing medication use assessments, and consulting with cardiologists to resolve inappropriate prescribing. | Average number of drugs prescribed per encounter  % encounters with antibiotics prescribed  % encounters with injection prescribed  % of drugs prescribed by generic  % of drugs prescribed from the essential drug list | Average number of drugs prescribed per encounter: Intervention vs routine practice 5.98 ± 1.82 vs 6.87± 2.4, (p=0.043, paired t-test) % encounters with antibiotics  29 (60.4%) vs 38 (79.2%), (p=0.009, chi-square test) % encounters with injection  36 (75.0%) vs 35 (73.0%), (p=0.496, chi-square test)  % of drugs prescribed by generic name  279 (97.2%) vs 238 (72.1%), (p=0.002, chi-square test) % of drugs prescribed from the essential drug  285 (99.3%) vs314 (95.2%), (p=0.152, chi-square test) |
| Eltayeb  2005 [39],  Sudan | cRCT | Government health centers | Physicians | 2 | 450 | 150 | A&F,  A&F + seminars + Prescribing guidelines,  No Intervention (Control) A&F + AD + prescribing guideline | None | Number of inappropriate prescriptions according to diagnosis  A&F vs no intervention  MD 3.8 (95% CI -1.2, 8.8) A&F + Seminar vs no intervention  MD 11.6 (95% CI 6.6 to 16.7) A&F + AD vs No intervention:  MD 14.2 (95% CI 9.2 to 19.2)  Prescriptions with inappropriate dose and/or duration of therapy A&F vs no intervention  MD 3.2 (95% CI 0.3, 6.1) A & F + Seminar vs no intervention  MD 9.6 (95% CI 6.7, 12.5) A&F + AD vs no intervention  MD 11.2 (95% CI 8.3,14.1) |
| Esmaily  2010 [37] ,  Iran | cRCT | GPs clinics | GPs | 3 | 5428 | 8052 | Educational training  Self-learning educational materials | Average number of drugs prescribed per encounter  % encounters with antibiotics prescribed  % encounters with injection prescribed | Average number of drugs per encounter  MD -0.2 (95% CI -0.7, 0.32) % encounters with antibiotics  Intervention vs control  (63% vs 60%) % encounters with injection  MD - (52% vs 58%) |
| Garjani  2009 [48]  Iran | RCT | public and private physicians’ clinic | Physician | 1 | 1135 | 1084 | Interactive group discussion on rational prescribing & Routine practices | Average number of drugs prescribed per encounter  % encounters with antibiotics prescribed  % encounters with injection prescribed  % of drugs prescribed by generic  % of drugs prescribed from the essential drug list | Average number drugs prescribed per encounter,  intervention vs control.  3.71% vs 3.76%  % of encounter with antibiotic   (38.9% vs 37.2%)  % encounter with injection  (57.7% vs 54.8%) |
| Hasan  1997 [41],  Sharjah, UAE | Pre and Post | Primary Health Care Centers | Physician | 1 | 6000 | 6000 | ACC guidelines | Average number of drugs prescribed per encounter  % encounters with antibiotics prescribed  % encounters with injection prescribed  % of drugs prescribed by generic  % of drugs prescribed from the essential drug list | Average number of drugs prescribed per encounter:  Pre vs Post: (2.8 vs 2.7) % of encounters with an antibiotic  (45% vs 35%)  % of encounters with an injection (16% vs 14%)  % of drugs prescribed from the PHC drug pharmacy list  (100% vs 100%)  % of drugs prescribed by generic name  (0% vs 0%) |
| Kandeel  2019 [40],  Egypt | Pre & Post | Primary healthcare centers, hospitals, private pharmacies | Physicians, pharmacists, and public | 5 | 607 | 331 | Educational material,  5-days Training course,  Communication and Social media campaign using Facebook and YouTube channel | % of encounters with an antibiotic prescribed | % of encounters with antibiotics  Pre vs post intervention  83.7% vs 64% |
| Ibrahim 2012 [44],  Egypt | Pre and Post | Physicians | Physicians | 1 | 600 | 600 | Distributing antibiotic guidelines and holding workshops activities directed towards rational drug use. | Average number of drugs prescribed per encounter  % encounters with an antibiotics  % encounters with an injection  % of drugs prescribed by generic  % of drugs prescribed from the essential drug list | Average number of drugs prescribed per encounter, Pre vs Post: 2.7 vs 2.5 % of encounters with antibiotic,  (47% vs Post 34%) % of encounters with injection:  (18% vs 16%) % of Drugs prescribed by generic name:  (0% vs 0%)  % of drugs prescribed from PHC drug formulary 100% vs 100% |
| Nejad  2016 [38],  Iran | RCT | Physicians | General practitioners | 7 | 485 | 421 | Feedback through TPLs Feedback through STM | None | DDD post intervention:  TPL vs Control:  104.38 (95% CI 84.19 to 124.57) vs  156.17 (95% CI 139.61,172.73)  STM vs Control:  101.9 (95% CI 86.47 to 117.34) vs  156.17 (95% CI 139.61 to172.73) |
| Soleymani 2019 [35],  Iran | RCT | Outpatients' clinic | Physicians | 3 | 601 | 208 | RA&F NA&F PEM | Average number of drugs prescribed per encounter  % encounters with an antibiotic prescribed  % encounters with an injection prescribed | Average number of drugs per prescriptions  RA & F vs Control:  2.93 (95% CI 2.84 to 3.01) vs  2.92 (95% CI 2.83 to 3.02)  NA & F vs Control:  2.86 (95% CI 2.78 to 2.93) vs  2.92 (95% CI 2.83 to 3.02)  PEM vs Control:  2.89 (95% CI 2.81, 2.93) vs  2.92 (95% CI 2.83 to 3.02)  % of encounter with antibiotics:  RA&F vs Control: 44.7% vs 40.9%  NA&F vs Control: 42.3% vs 40.9%  PEM vs Control: 43.9% vs 40.9%  % of encounter with injection:  RA&F vs Control: 39.2% vs 40.7%  NA&F vs Control: 38.8% vs 40.7%  PEM vs Control: 37.7% vs 40.7% |
| Salehi 2025  Iran[46] | Pre and Post | Hospital outpatients Emergency department | Physicians | 2 weeks | 235 | 314 | 20-minute educational video regarding appropriate indication, dose, and duration of antibiotics | % encounters with an antibiotic prescribed | % of encounter with antibiotics:  Intervention vs control:  42.6% vs 31.9% (p<0.001, chi-square test)  Appropriate indication of antibiotics:  81.3% vs 97.3% (p<0.0001, chi-square test) |
| Elnajjar 2025  UAE [43] | Quasi-experimental | Tertiary care hospital | Physicians | 6 | NA | NA | ASP |  | Mean DDD Pre vs Post ASP: 122 per 1000 Patient days vs  86 DDD per 1000 Patient days (p=0.001, paired t -test), 29% reduction |
| Gulam 2025  UAE [42] | Quasi-experimental | Hospital | Physicians | 11 | 260 | 237 | Pharmacist-led prospective audit and feedback |  | Length of therapy  Intervention vs Control:  Median (IQR): 3(2.5-5) vs 4 (3-6), (p=<0.001, Mann–Whitney U test)  Days of therapy Median (IQR): 4 (3-6.5) vs 5(3-9), (p=0.001, Mann–Whitney U test) |
| Notes:  **AB** – Antibiotic ; **ACC** – Antibiotic Control Committee; **A&F** – Audit and Feedback; **AMR** – Antimicrobial Resistance;  **ASP** – Antimicrobial Stewardship Program; **DDD** – Defined Daily Dose; **Edu** – Educational; **GL** – Guideline; **Med** – Medication; **NA&F** – New-design Audit and Feedback; **PEM** – Printed Educational Materials; **RA&F** – Routine Audit and Feedback; **STM** – Short Text Messages; **TPLs** – Traditional Postal Letters; PHC – Primary Health Care  **MD** – Mean Difference; SD – Standard Deviation; **CI** – Confidence Interval; **cRCT** – Cluster Randomized Controlled Trial; **RCT** – Randomized Controlled Trial  **Outcomes used in meta-analysis:** Mean number of drugs prescribed per encounter; % encounters with an antibiotic; % encounters with an injection  **Outcomes used in narrative analysis:** % of drugs prescribed by generic; % of drugs prescribed from the essential drug list; percentage of encounters with appropriate antibiotics | | | | | | | | | |

**Supplementary Table 2: GRADE assessment**

| **Summary of findings:** | | | | | | |
| --- | --- | --- | --- | --- | --- | --- |
| **Multifaceted interventions compared to no intervention for prescribing indicators** | | | | | | |
| **Patient or population:** decreasing the mean number of drugs per prescription  **Setting:**  **Intervention:** Multifaceted interventions  **Comparison:** no intervention | | | | | | |
| Outcomes | **Anticipated absolute effects^*^** (95% CI) | | Relative effect (95% CI) | № of participants (studies) | Certainty of the evidence (GRADE) | Comments |
|  | **Risk with no intervention** | **Risk with Multifaceted interventions** |  |  |  |  |
| Mean number of drugs on prescription (mean number of drugs) | - | WMD **0.1 SD lower** (-0.18 lower to 0.02 higher) | - | (8 non-randomized studies) | ⨁⨁◯◯ Low |  |
| Percentage of Antibiotics | 441 per 1,000 | **339 per 1,000** (448 to 244) | **OR 0.65** (0.41 to 1.03) | 9656 (13 non-randomized studies) | ⨁◯◯◯ Very low |  |
| Percentage of Injections | 403 per 1,000 | **365 per 1,000** (333 to 396) | **OR 0.85** (0.74 to 0.97) | 30220 (8 non-randomized studies) | ⨁◯◯◯ Very low |  |
| ***The risk in the intervention group** (and its 95% confidence interval) is based on the assumed risk in the comparison group and the **relative effect** of the intervention (and its 95% CI). **CI:** confidence interval; **OR:** odds ratio; **SMD:** standardized mean difference | | | | | | |

| **GRADE Working Group grades of evidence** **High certainty:** we are very confident that the true effect lies close to that of the estimate of the effect. **Moderate certainty:** we are moderately confident in the effect estimate: the true effect is likely to be close to the estimate of the effect, but there is a possibility that it is substantially different. **Low certainty:** our confidence in the effect estimate is limited: the true effect may be substantially different from the estimate of the effect. **Very low certainty:** we have very little confidence in the effect estimate: the true effect is likely to be substantially different from the estimate of effect. |
| --- |

**Outcome 1: Mean number of drugs per encounter**


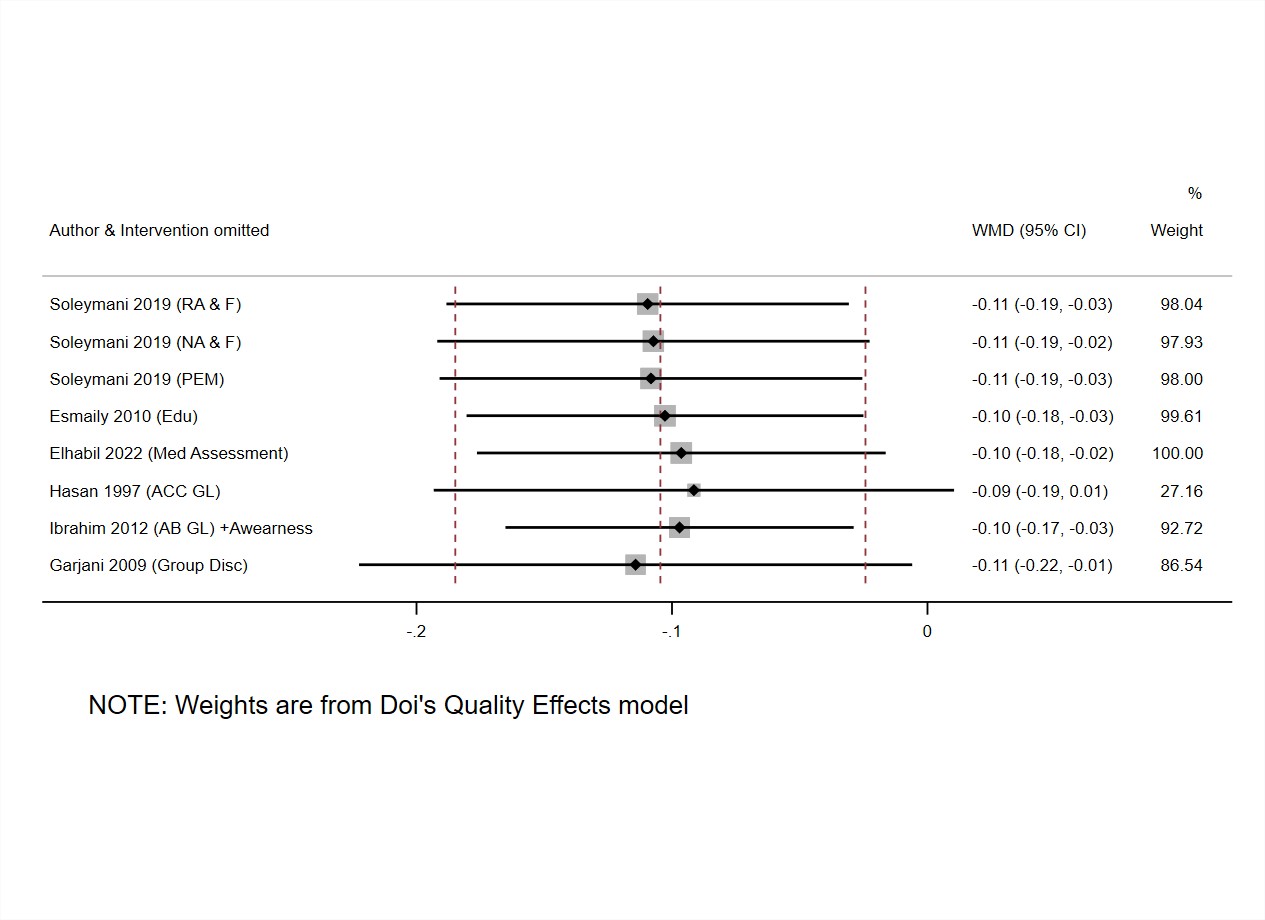


Supplementary Figure 1: Sensitivity analysis of the effectiveness of multifaceted and educational interventions on the mean number of drugs per encounter

**Publication bias**


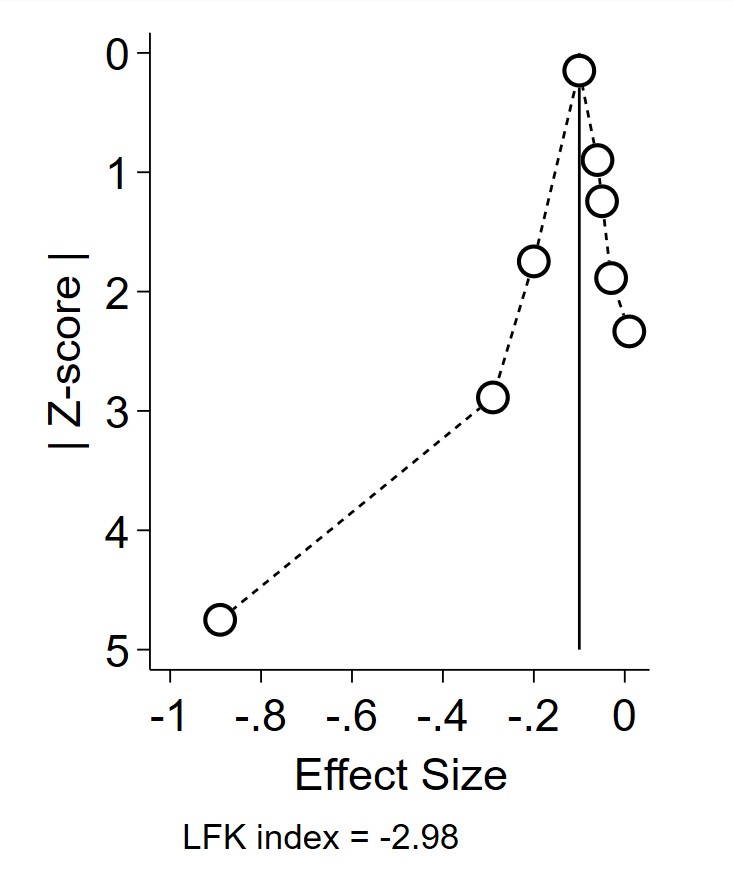


Supplementary Figure 2: DOI plot & LFK index for the mean number of drugs per encounter

**Supplementary Table 3: Sub-group analysis of the effectiveness of multifaceted and educational interventions on the mean number of drugs per encounter**

| **Sub-groups** | **No. of studies** | **N Intervention** | **N Control** | **Mean Difference**  **(95% CI)** | **I^2^ (%)** | **P *_effect_*** | **P *_subgroup​_*** |
| --- | --- | --- | --- | --- | --- | --- | --- |
| Overall pooled estimate | 8 | 13812 | 16408 | -0.10 (-0.18, -0.02) | 99.8% | <0.001 | NA |
| Intervention types |  |  |  |  |  |  | 0.891 |
| Multifaceted | 4 | 6448 | 6464 | -0.11 (-0.21, -0.01) | 99.8% | <0.001 |  |
| Education only | 4 | 7364 | 9944 | -0.10 (-0.23, -0.0) | 99.9% | <0.001 |  |
| Study settings |  |  |  |  |  |  | 0.738 |
| Primary care clinics | 6 | 13164 | 15760 | -0.09 (-0.16, -0.02) | 99.7% | <0.001 |  |
| Hospitals | 2 | 648 | 648 | -0.21 (-0.94, 0.52) | 60% | 0.112 |  |
| Study design |  |  |  |  |  |  | 0.333 |
| RCT | 5 | 7164 | 9760 | -0.05 (-0.12, 0.03) | 99.5% | <0.001 |  |
| Non-RCT/Quasi-exp | 3 | 6648 | 6648 | -0.12 (-0.24, 0.00) | 99.9% | 0.003 |  |
| Study region |  |  |  |  |  |  | 0.869 |
| GCC countries | 1 | 6000 | 6000 | -0.10 (-0.10, -0.10) | 0% | <0.001 |  |
| Other MENA countries | 7 | 7812 | 10408 | -0.09 (-0.19, 0.01) | 99.8% | <0.001 |  |

**P *_effect:_*** P value of the intervention’s effect within that specific subgroup. **P *_subgroup_*:** P value for the 𝜒2 (Chi-squared) test of differences *between* the subgroups; **Regional Categorization: GCC countries:** Includes studies conducted in Saudi Arabia, United Arab Emirates, Qatar, Kuwait, Oman, and Bahrain. **Other MENA countries:** Includes studies from other Middle East and North Africa countries (e.g., Jordan, Egypt, Iran, Lebanon, Palestine). **Intervention Type:** "**Multifaceted**" interventions using two or more distinct strategies (e.g., education plus audit and feedback), while "**Education only**" refers to single-component educational programs. **Study Design:** RCT: Randomized Controlled Trial; Non-RCT: Includes quasi-experimental, pre-post studies, and non-randomized controlled trials.

**Abbreviations:** CI: Confidence Interval; MD: Mean Difference; OR: Odds Ratio; GCC: Gulf Cooperation Council; MENA: Middle East and North Africa; **NA**: Not Applicable.

**Outcome 2: Effectiveness of multifaceted and educational interventions on the percentage of encounters with an antibiotic prescribed**


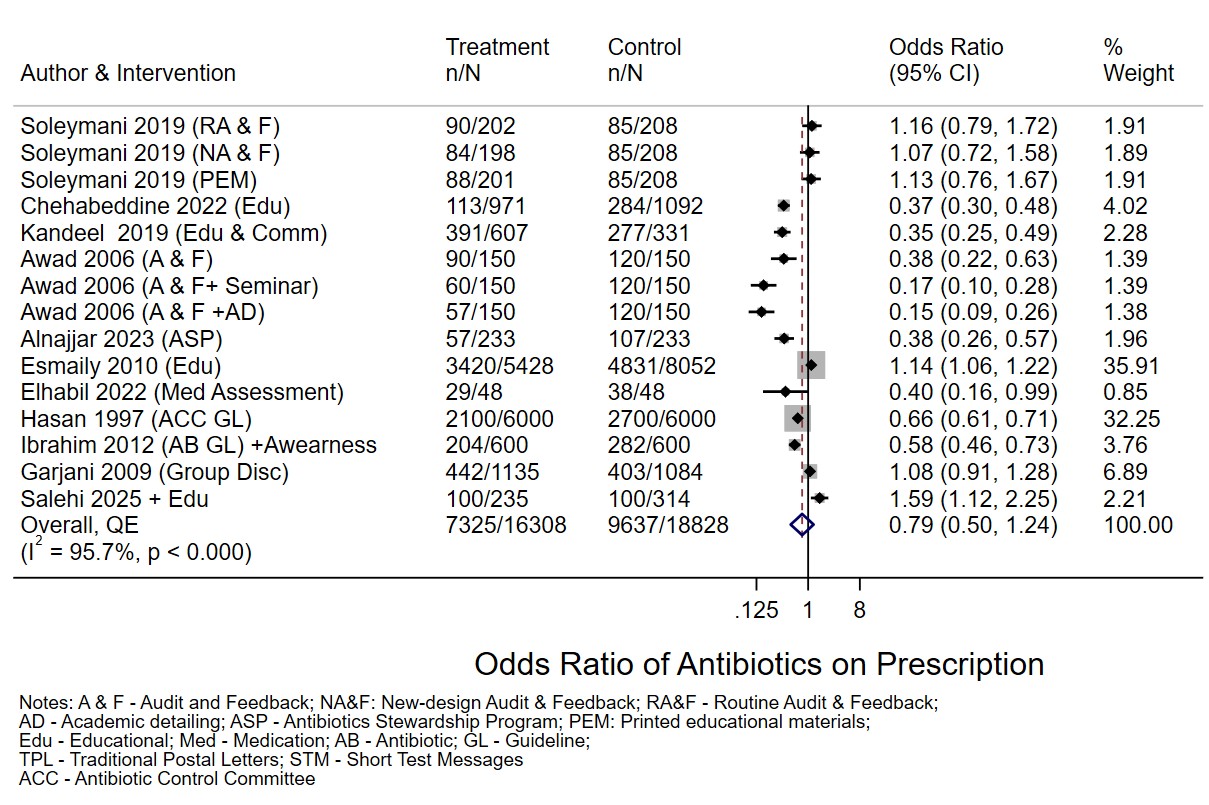


Supplementary figure 3: Forest plot of the effectiveness of multifaceted and educational interventions on the percentage of encounters with an antibiotic prescribed (Before sensitivity analysis)

**
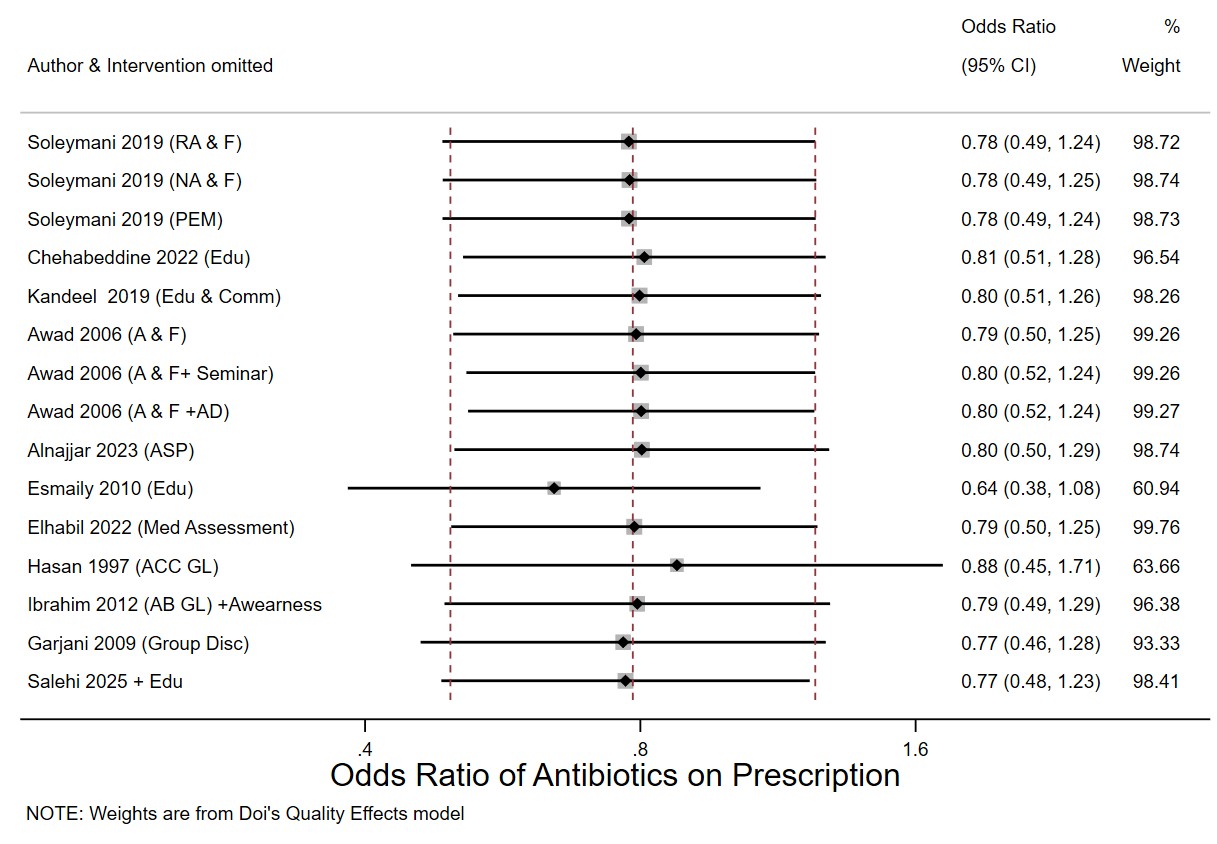
**

Supplementary Figure 4: Sensitivity analysis of the effectiveness of multifaceted and educational interventions on the percentage of encounters with an antibiotic prescribed

**Publication Bias:**

**
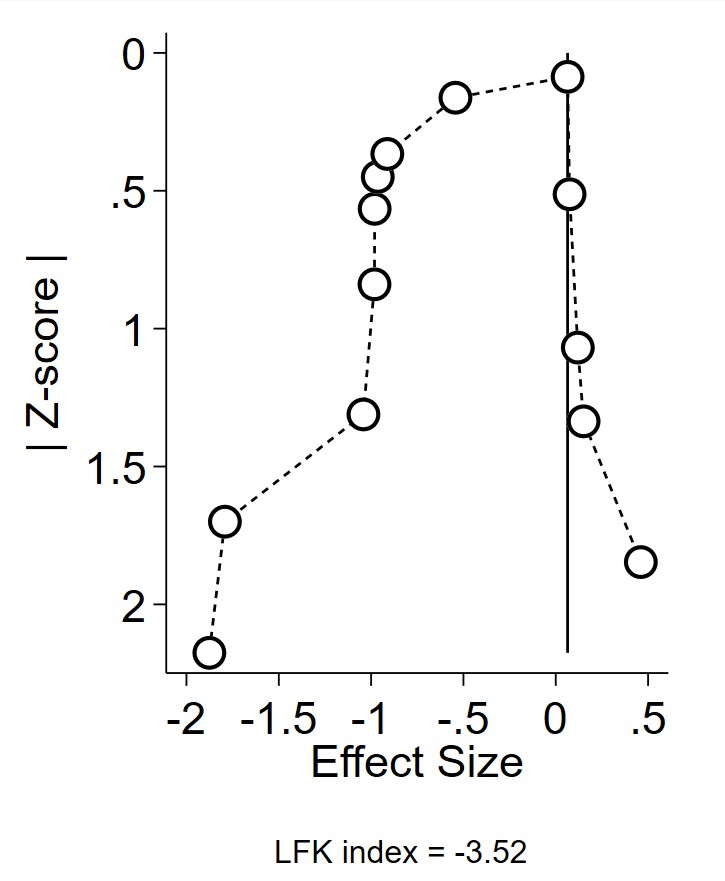
**

Supplementary figure 5: Doi plot and LFK index for the percentage of encounters with an antibiotic prescribed

**Supplementary Table 4: Subgroup analysis of the effectiveness of multifaceted and educational interventions on the percentage of encounters with an antibiotic prescribed**

| **Sub-groups** | **No. of studies** | **N Interverntion** | **N Control** | **Odds Ratio (95% CI)** | **I^2^ (%)** | **P*_effect​_*** | **P *_subgroup​_*** |
| --- | --- | --- | --- | --- | --- | --- | --- |
| **Overall pooled estimate** | **13** | **1805** | **2106** | **0.65 (0.41, 1.03)** | **93.5%** | **<0.001** | **NA** |
| **Intervention types** |  |  |  |  |  |  | **0.332** |
| Multifaceted | 7 | 467 | 675 | 0.48 (0.24, 0.93) | 91.7% | <0.001 |  |
| Education only | 6 | 1338 | 1431 | 0.73 (0.43, 1.25) | 91.7% | <0.001 |  |
| **Study settings** |  |  |  |  |  |  | **0.936** |
| Primary care clinics | 9 | 1415 | 1579 | 0.64 (0.34, 1.22) | 91.1% | <0.001 |  |
| Hospitals | 4 | 390 | 527 | 0.67 (0.32, 1.39) | 91.1% | <0.001 |  |
| **Study design** |  |  |  |  |  |  | **0.738** |
| RCT | 8 | 781 | 804 | 0.69 (0.34, 1.39) | 91.1% | <0.001 |  |
| Non-RCT/Quasi-exp | 5 |  |  | 0.59 (0.32, 1.08) | 96.0% | <0.001 |  |
| **Study region** |  |  |  |  |  |  | **0.079** |
| GCC countries | 1 | 57 | 107 | 0.38 (0.26, 0.57) | 95.0% | <0.001 |  |
| Other MENA countries | 12 | 1748 | 1999 | 0.67 (0.41, 1.10) | 93.5% | <0.001 |  |

**P *_effect:_*** P value of the intervention’s effect within that specific subgroup. **P *_subgroup_*:** P value for the 𝜒2 (Chi-squared) test of differences *between* the subgroups; **Regional Categorization: GCC countries:** Includes studies conducted in Saudi Arabia, United Arab Emirates, Qatar, Kuwait, Oman, and Bahrain. **Other MENA countries:** Includes studies from other Middle East and North Africa countries (e.g., Jordan, Egypt, Iran, Lebanon, Palestine). **Intervention Type:** "**Multifaceted**" interventions using two or more distinct strategies (e.g., education plus audit and feedback), while "**Education only**" refers to single-component educational programs. **Study Design:** RCT: Randomized Controlled Trial; Non-RCT: Includes quasi-experimental, pre-post studies, and non-randomized controlled trials.

**Abbreviations:** CI: Confidence Interval; MD: Mean Difference; OR: Odds Ratio; GCC: Gulf Cooperation Council; MENA: Middle East and North Africa; **NA**: Not Applicable

**Outcome 3**: **Effectiveness of multifaceted and educational interventions on the percentage of encounters with an injection**


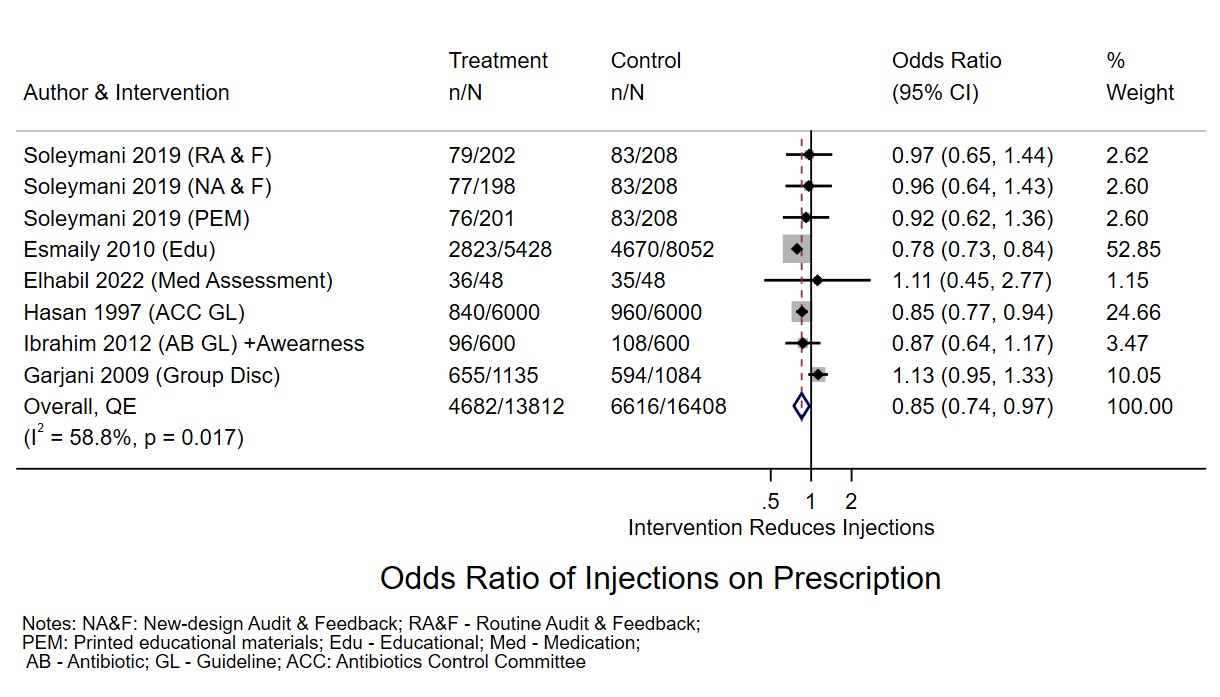


Supplementary figure 6: Forest plot of the effectiveness of multifaceted and educational interventions on the percentage of encounters with an injection prescribed (before sensitivity analysis)


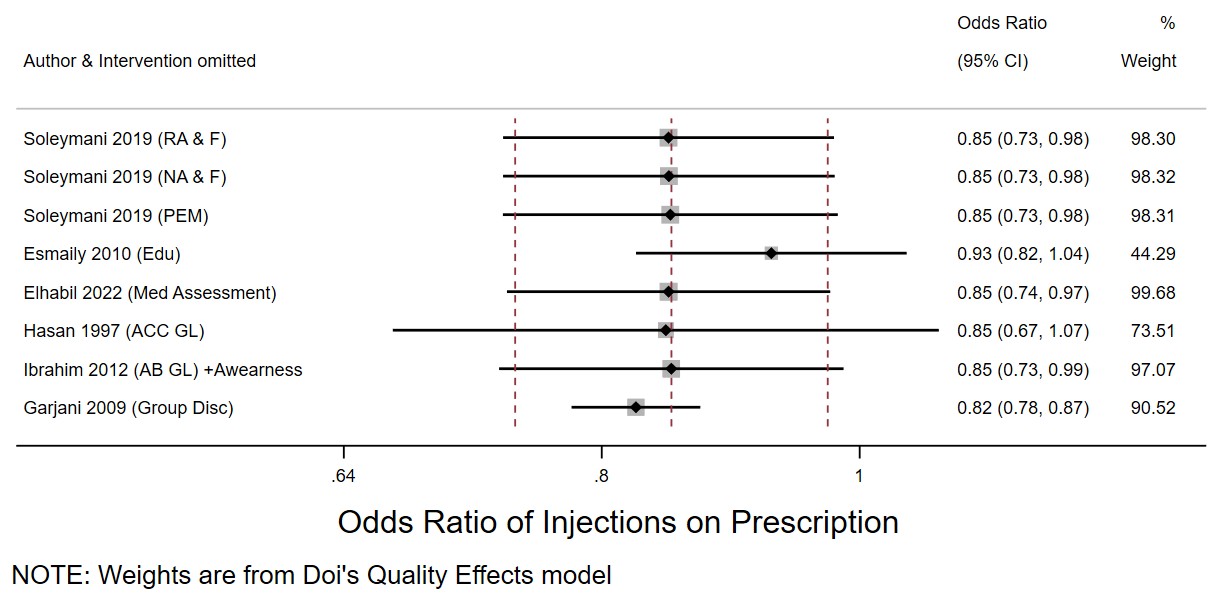


Supplementary Figure 7: Sensitivity analysis of the effectiveness of multifaceted and educational interventions on the percentage of encounters with an injection prescribed

**
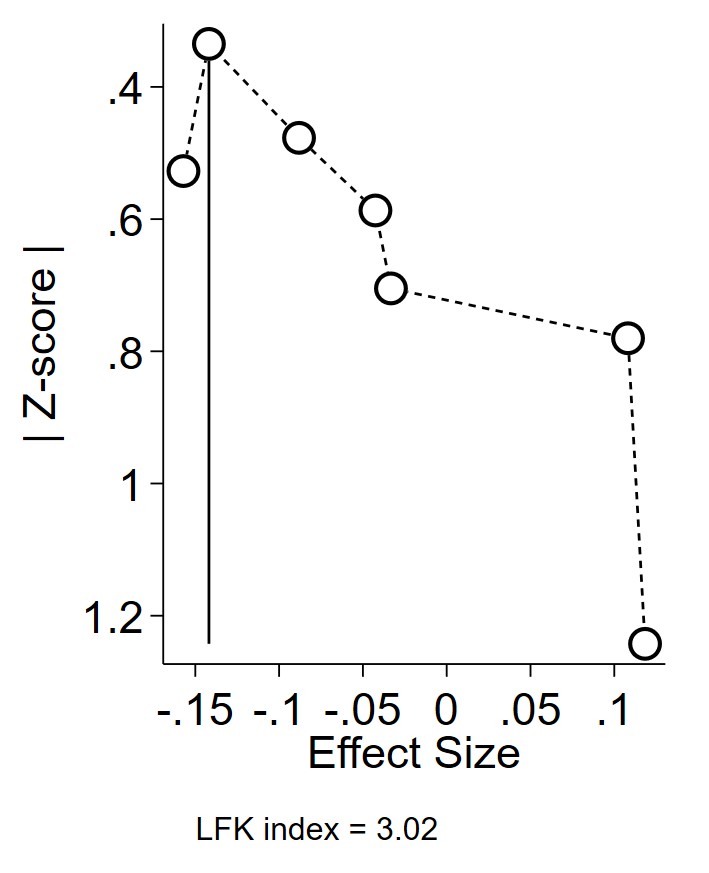
**

Supplementary figure 8: DOI & LFK index Plot for the percentage of encounters with an injection prescribed

**Supplementary Table 5: Subgroup analysis of the effectiveness of multifaceted and educational interventions on the percentage of encounters with an injection prescribed**

| **Sub-groups** | **No. of studies** | **N Interverntion** | **N Control** | **Odds Ratio (95% CI)** | **I^2^ (%)** | **P *_effect_*​** | **P *_subgroup​_*** |
| --- | --- | --- | --- | --- | --- | --- | --- |
| **Overall pooled estimate** | **7** | **1859** | **1946** | **0.93 (0.82, 1.04)** | **25%** | **0.236** | **NA** |
| **Intervention types** |  |  |  |  |  |  | **0.102** |
| Multifaceted | 4 | 1032 | 1161 | 0.88 (0.80, 0.97) | 0% | 0.824 |  |
| Education only | 3 | 827 | 785 | 1.04 (0.87, 1.24) | 0% | 0.824 |  |
| **Facility types** |  |  |  |  |  |  | **0.817** |
| Primary care clinics | 5 | 1727 | 1803 | 0.93 (0.79, 1.09) | 0% | 0.610 |  |
| Hospitals | 2 | 132 | 143 | 0.89 (0.67, 1.19) | 0% | 0.610 |  |
| **Study design** |  |  |  |  |  |  | **0.016** |
| RCT | 4 | 887 | 843 | 1.06 (0.92, 1.21) | 0% | 0.849 |  |
| Non-RCT/Quasi-exp | 3 | 972 | 1103 | 0.86 (0.78, 0.95) | 0% | 0.849 |  |
| **Study region** |  |  |  |  |  |  | **0.024** |
| GCC countries | 1 | 840 | 960 | 0.85 (0.77, 0.94) | 0% | 0.715 |  |
| Other MENA countries | 6 | 1019 | 986 | 1.03 (0.91, 1.16) | 0% | 0.715 |  |

**P *_effect:_*** P value of the intervention’s effect within that specific subgroup. **P *_subgroup_*:** P value for the 𝜒2 (Chi-squared) test of differences *between* the subgroups; **Regional Categorization: GCC countries:** Includes studies conducted in Saudi Arabia, United Arab Emirates, Qatar, Kuwait, Oman, and Bahrain. **Other MENA countries:** Includes studies from other Middle East and North Africa countries (e.g., Jordan, Egypt, Iran, Lebanon, Palestine). **Intervention Type:** "**Multifaceted**" interventions using two or more distinct strategies (e.g., education plus audit and feedback), while "**Education** **only**" refers to single-component educational programs. **Study Design:** RCT: Randomized Controlled Trial; Non-RCT: Includes quasi-experimental, pre-post studies, and non-randomized controlled trials.

**Abbreviations:** CI: Confidence Interval; MD: Mean Difference; OR: Odds Ratio; GCC: Gulf Cooperation Council; MENA: Middle East and North Africa; **NA**: Not Applicable.
